# Exercise‐induced effects on atherogenesis and tryptophan catabolism via the kynurenine pathway in an HIV‐associated atherosclerosis mouse model

**DOI:** 10.1113/EP092744

**Published:** 2025-08-25

**Authors:** Marcus V. S. Rangel, Mohammad Afaque Alam, Mohammad Islamuddin, Zheng Chen, Xuebin Qin, Juliana P. Borges

**Affiliations:** ^1^ Tulane National Primate Research Center Covington Louisiana USA; ^2^ Tulane University School of Medicine New Orleans Louisiana USA; ^3^ Laboratory of Physical Activity and Health Promotion Institute of Physical Education and Sports, University of Rio de Janeiro State Rio de Janeiro Rio de Janeiro Brazil

**Keywords:** acquired immune deficiency syndrome, atherosclerosis, exercise training, metabolism

## Abstract

A mouse model of HIV‐associated atherosclerosis (*Tg26* ^+/−^ *ApoE* ^−/−^) exhibited increased plaque area compared with the *ApoE* ^−/−^ mouse, linked to elevated indoleamine 2,3‐dioxygenase (IDO) activity. IDO catalyses the conversion of tryptophan (TRP) into kynurenine (KYN), measured by the KYN‐to‐TRP ratio. As a biomarker of inflammation, IDO has been implicated as a risk factor for cardiovascular disease. To investigate the effect of exercise training on atherogenesis and IDO activity in *Tg26* ^+/−^ *ApoE* ^−/−^ mice, nine *Tg26* ^+/−^ *ApoE* ^−/−^ and 18 *ApoE* ^−/−^ male mice were fed an atherogenic diet and randomized into exercised or control groups. The exercised groups underwent an 8‐week treadmill protocol at moderate intensity (five times per week at 60% maximum velocity). Concentrations of KYN, TRP and cytokines were measured using ELISA, immune expression by flow cytometry, and lipid profile by a biochemistry analyser. Aortas were harvested post mortem for *en face* analysis. *Tg26* ^+/−^ *ApoE* ^−/−^ mice showed ∼40% larger plaques than *ApoE* ^−/−^ mice (*P* = 0.01), with slightly higher neutrophil (*P* = 0.05) and monocyte expression (*P* = 0.06). Plaque area was reduced by 40% in exercised *ApoE* ^−/−^ mice (*P* = 0.04), but by only 12% in exercised *Tg26* ^+/−^ *ApoE* ^−/−^ animals (*P* = 0.85). Exercised *Tg26* ^+/−^ *ApoE* ^−/−^ mice showed higher IDO activity than exercised *ApoE* ^−/−^ mice (58.57% ± 6.88% vs. −4.62% ± 17.20%, *P* = 0.01), which was positively correlated with plaque area (*R* = 0.99, *P* = 0.02). Exercised *ApoE*−^/^− mice showed significantly lower triglyceride levels compared with exercised *Tg26* ^+/−^ *ApoE* ^−/−^ mice (75.8 ± 14.8 vs. 165.2 ± 43.6 mg/dL; *P* = 0.02). Unlike *ApoE* ^−/−^ mice, moderate‐intensity aerobic training did not reduce plaque area in mice with HIV‐associated atherosclerosis. Moreover, exercise training appeared to increase inflammation in *Tg26* ^+/−^ *ApoE* ^−/−^ mice, as indicated by elevated IDO activity.

## Abstract (highlights)

- **What is the central question of this study?**
  Does moderate‐intensity aerobic exercise training influence the development of human immunodeficiency virus (HIV)‐associated atherosclerosis and tryptophan metabolism via the kynurenine pathway in an HIV‐associated atherosclerosis mouse model?
- **What is the main finding and its importance?**
  Moderate‐intensity aerobic exercise reduced atherosclerotic plaque size in *ApoE* ^−/−^ mice, but not in HIV‐transgenic mice, in which it was associated with elevated indoleamine 2,3‐dioxygenase activity. It is possible that HIV‐associated inflammation might attenuate the usual benefits of exercise on atherosclerosis, highlighting the need for further investigation and tailored exercise recommendations for people living with HIV.

## 1 INTRODUCTION

In the combined antiretroviral therapy era, atherosclerotic cardiovascular diseases (ASCVD), such as myocardial infarction and stroke, have become major concerns for people living with human immunodeficiency virus (HIV) (GBD 2019 Stroke Collaborators, 2021; Hsue & Waters, 2019). People living with HIV face a 1.5–3 times greater risk of developing ASCVD (Feinstein et al., 2019), which has garnered significant attention from the scientific community. Until recently, research on HIV‐associated atherosclerosis was limited to clinical and epidemiological studies. However, understanding the mechanisms involved in the atherogenic process still necessitates the use of animal models (Cardinot et al., 2016).

A new mouse model of HIV‐associated atherosclerosis, named *Tg26* ^+/−^ *ApoE* ^−/−^, was recently developed by crossing transgenic *Tg26* ^+/−^ mice, which express HIV‐1 transcripts, with apolipoprotein E‐deficient (*ApoE* ^−/−^) mice (Kearns, Liu, Dai, et al., 2019; Kearns, Velasquez, Liu, et al. (2019). Kearns, Velasquez, Liu, et al. (2019) observed that compared with *ApoE* ^−/−^ mice, the *Tg26* ^+/−^ *ApoE* ^−/−^ mice exhibited accelerated HIV‐associated atherosclerosis. This was linked to overactivation of the kynurenine (KYN) pathway, evidenced by increased indoleamine 2,3‐dioxygenase (IDO) activity (KYN/tryptophan ratio). The KYN pathway, responsible for degrading the essential amino acid tryptophan (TRP), is orchestrated by cytokine production, and its metabolites regulate inflammation (Baumgartner et al., 2019). KYN has recently been identified as a significant predictor of cardiovascular events and atherosclerosis progression in the general population (Zhang et al., 2022) and among people living with HIV (Qi et al., 2018). The establishment of this new model represented a landmark in understanding how HIV infection accelerates atherogenesis, opening avenues for development of therapeutic strategies to reduce the risk of HIV‐associated ASCVD.

Exercise has consistently been shown to prevent and manage ASCVD in the general population (Meyer‐Lindemann et al., 2023). Investigating this effect in the HIV context is crucial owing to the accelerated atherosclerosis and overactivity of IDO observed in HIV settings (Kearns, Velasquez, Liu, et al., 2019). To date, no studies have examined the effects of supervised exercise training on the development of HIV‐associated atherosclerosis or on exercise‐induced changes in the KYN pathway. Evidence in this area could help to identify new therapeutic approaches to combat HIV‐associated ASCVD. Therefore, our primary aim was to investigate the effect of aerobic exercise training on atherogenesis and TRP catabolism via the KYN pathway in *Tg26* ^+/−^ *ApoE* ^−/−^ mice.

## 2 MATERIALS AND METHODS

### 2.1 Ethical approval

The experiments were approved by the Animal Care and Use Committee at Tulane University School of Medicine (protocol ID 1499) and were conducted according to the *Guide for the Care and Use of Laboratory Animals* (US National Institutes of Health Guide, 8th edition, 2011). All animal experiments adhered to the ARRIVE guidelines 2.0 and to the policies of *Experimental Physiology* regarding animal experimentation.

### 2.2 Animals

*ApoE* ^−/−^ mice were obtained from the Jackson Laboratory and crossed with *Tg26* ^+/−^ mice with a B6 background to generate *Tg26* ^+/−^ *ApoE* ^−/−^ mice, as previously described (Kearns, Velasquez, Liu, et al., 2019). Genotyping was performed via tail preparation and PCR to confirm the presence of the HIV transgene in *Tg26* ^+/−^ *ApoE* ^−/−^ mice using specific primer pairs (forward: 5′‐TCCAGTTTGGAAAGGACCAG‐3′; reverse: 5′‐TTGCCACACAATCATCACCT‐3′), as previously described (Kearns, Velasquez, Liu, et al., 2019). All animals were housed in an animal facility at Tulane University School of Medicine (New Orleans, LA, USA), under a 12 h–12 h light–dark cycle in a temperature‐controlled environment (22°C), with food and water provided ad libitum.

### 2.3 Experimental design

**FIGURE 1:**
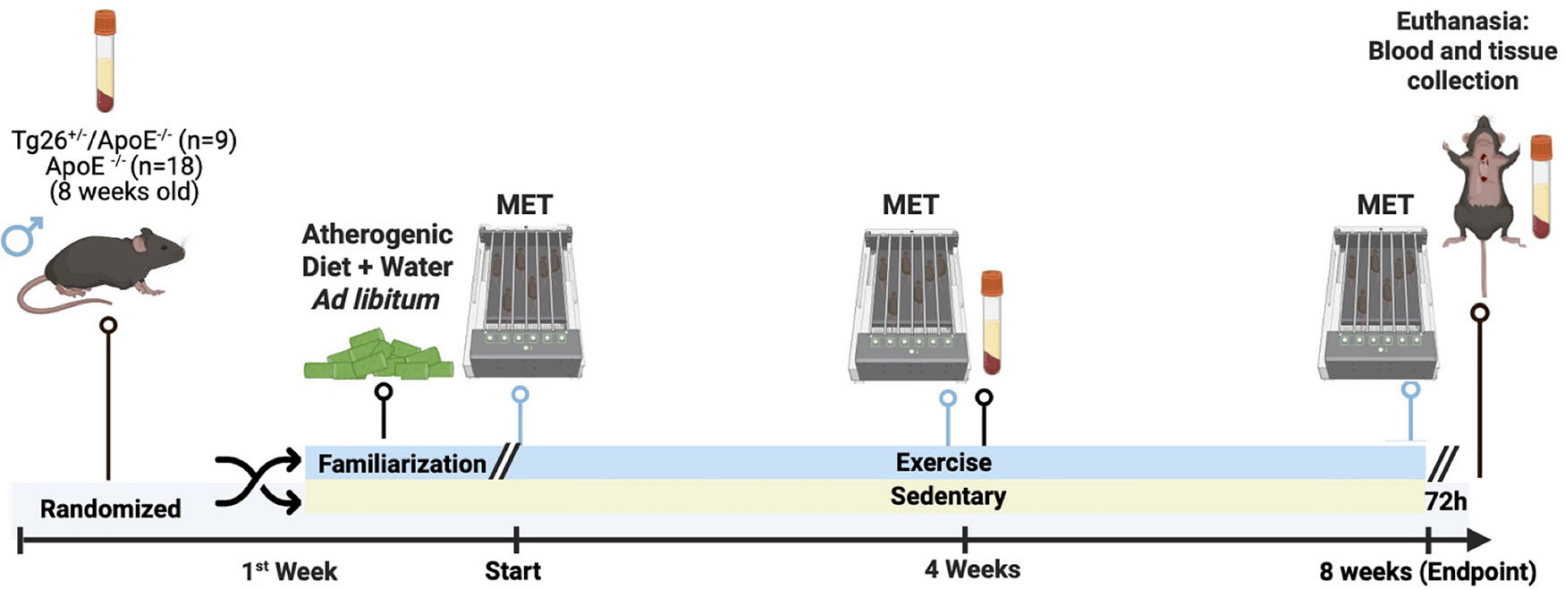
Representative schematic diagram of the study protocol. Black lines indicate procedures performed in all groups, whereas blue lines indicate procedures performed only in the exercised groups. Created with BioRender.com. Abbreviation: MET, maximal exercise test.

Figure 1 presents a representative schematic diagram of the study protocol. Male 8‐week‐old *Tg26* ^+/−^ *ApoE* ^−/−^ (*n* = 9) and *ApoE* ^−/−^ (*n* = 18) mice were fed an atherogenic diet (D12108C; Research Diets, Inc., New Brunswick, NJ, USA) containing 20.1% saturated fat, 1.37% cholesterol and 0% sodium cholate for 8 weeks. Concurrently with the initiation of the diet, the mice were randomized into sedentary (*Tg26* ^+/−^ *ApoE* ^−/−^, *n* = 4; and *ApoE* ^−/−^, *n* = 9) or exercised (*Tg26* ^+/−^ *ApoE* ^−/−^ Ex, *n* = 5; and *ApoE* ^−/−^ Ex, *n* = 9) groups. The exercised groups underwent an 8‐week treadmill protocol at moderate intensity (five times per week at 60% maximum velocity). Blood was collected via tail bleeding at baseline and the fourth week. Seventy‐two hours after the final exercise session, all animals were killed by exsanguination under isoflurane anaesthesia administered in an induction chamber. Death was ensured by cervical dislocation. Subsequently, a thoracotomy was performed to allow for the complete excision of the aorta, and the visceral fat pads (epididymal, retroperitoneal and inguinal) were harvested for analysis of body composition.

### 2.4 Maximal exercise test and exercise training

Initially, all animals were adapted to a motor‐driven rodent treadmill (AVS projects, São Paulo, Brazil) at a low velocity (12 m/min, 0% inclination, 15 min/day, for 3 days consecutively). Maximum velocity (*V*_max_) was determined for each mouse in the exercised groups through a maximal exercise test conducted before, during (midpoint) and after the exercise training. The test began at 10 m/min, increasing by 1 m/min every 1 min until the animals reached exhaustion (Rodrigues et al., 2007; Stoyell‐Conti et al., 2019). Exhaustion was defined as the point at which they were unable to maintain their pace for ≥5 s, even when exposed to electrical stimuli (∼2 mA) from the treadmill, which caused minor discomfort but no harm.

Approximately 72 h after the initial maximal exercise test, the exercise training was conducted for 8 weeks on a motorized treadmill (AVS projects, São Paulo, Brazil) at moderate intensity (60% of *V*_max_). Training sessions lasted 1 h/day, 5 days/week, with a gradual increase in speed and duration as follows: 30 min at 50% of *V*_max_ in the first week, 50 min at 50% of *V*_max_ in the second week, 60 min at 50%–60% of *V*_max_ in the third week, and 60 min at 60% of *V*_max_ from the fourth to the eighth week.

### 2.5 Body composition assessment

Body mass was measured at baseline, after 4 weeks and at the end of the experiment (eighth week) using a digital scale with a sensitivity of 0.01 g (Ohaus, Parsippany, NJ, USA). Post mortem, retroperitoneal, epididymal and inguinal fat pads were harvested and weighed. The body adiposity index was calculated as the sum of the visceral fat pad weights divided by the body mass multiplied by 100 (Silva et al., 2023).

### 2.6 Whole aorta *en face* analysis

After harvesting the whole aorta from the mice, the tissue was fixed in 4% paraformaldehyde overnight, then washed in phosphate buffer and propylene glycol. The aorta was subsequently stained with Oil Red‐O (Sigma–Aldrich) to highlight morphological characteristics of the plaques. After staining, the aorta was cleaned, opened and pinned down. A digital camera (Canon Inc., Tokyo, Japan) was used to capture images of the entire aorta. The total pixel area for the aortas and plaques was measured by manual tracing using ImageJ (NIH, USA). Two independent trained technicians, blinded to the experimental groups, analysed the images. The percentage of plaque area in the aorta was calculated as follows: plaque area in aorta (%) = (plaque area/whole aortic area) × 100, as previously described (Kearns, Liu, Dai, et al., 2019).

### 2.7 Lipid profile analysis

Serum samples obtained from end‐point aliquots were analysed for total cholesterol and triglyceride concentrations by the Clinical Pathology Core Facility at the Tulane National Primate Research Center. The assays were performed using standardized enzymatic colorimetric methods on an automated clinical chemistry analyser, as described previously (Kearns, Velasquez, Liu, et al., 2019). All procedures followed the standard operating protocols of the Core Facility.

### 2.8 Kynurenine pathway, fresh blood immunophenotyping and cytokine analysis

Biochemical experiments were conducted using at least two biologically distinct replicates. A subset of mice was used to analyse KYN pathway metabolites. At baseline and midpoint (fourth week), 10% of the blood volume (100 µL for a 20 g mouse) was collected via tail bleeding. At the end‐point, 100 µL of blood obtained by cardiac puncture was also collected. The blood was allowed to clot for 30 min at room temperature, then centrifuged at 326 g for 10 min. The serum was separated into fresh tubes and stored for analysis. Serum concentrations of KYN and TRP were quantified using ELISA kits according to the manufacturer's instructions (Rocky Mountain Diagnostics, Colorado Springs, CO, USA).

Immunophenotyping of fresh blood and serum cytokine levels were measured only at the end of the experiment owing to limited blood volume. Serum cytokines were quantified using ELISA kits following the manufacturer's instructions (Proteintech, Chicago, IL, USA), with minimum detectable levels as follows: tumour necrosis factor‐α (TNF‐α, KE10002; 1.0 pg/mL), interleukin‐6 (IL‐6, KE10007; 3.8 pg/mL) and interferon‐γ (IFN‐γ, KE10001; 5.5 pg/mL).

Immunophenotyping of leucocyte subpopulations (monocytes and neutrophils) was conducted using flow cytometry. Initially, EDTA‐treated whole blood was incubated at room temperature to lyse the red blood cells, following the manufacturer's instructions (A1049201, Thermo Fisher, MA, USA). Afterwards, the cells were resuspended in FACS buffer. To identify surface expression patterns, fluorochrome‐labelled monoclonal antibodies [CD45 (eBioscience 48‐0451‐82), CD11b (eBioscience 25‐0112‐81), Ly6C (eBioscience 17‐5932‐82) and Ly6G (eBioscience 11‐5931‐82)] were added to the tubes and incubated for 30 min in the dark at room temperature. Cell sorting was performed at the Flow Cytometry Core Facility at Tulane University School of Medicine, and data analysis was conducted using FlowJo software (Tree Star, Ashland, OR, USA).

### 2.9 Statistical analysis

Data were tested for normality using the Shapiro–Wilk test and are presented as the mean ± SD. A priori sample size calculation was performed using G*Power v.3.1 software (Düsseldorf, NRW Germany), based on data from Kearns, Velasquez, Liu, et al. (2019), with the aorta plaque area defined as the primary outcome. The analysis considered a Cohen's *d* effect size of 1.35 (converted to *f* = 0.96), four experimental groups, a power of 80% and α = 0.05, resulting in an estimated required total sample size of *n* = 26. A *post hoc* power analysis using the observed data revealed an effect size of 1.24 and a statistical power of 97%.

Three‐way ANOVA was used for comparisons involving three factors (strain, intervention and time), such as body mass and KYN metabolites. Two‐way ANOVA was used for end‐point analysis outcomes (strain and intervention), such as aorta measurements, immune expression, lipid profile and cytokine analysis. Tukey's *post hoc* tests were used for multiple comparisons within and between the groups. Additionally, Pearson's correlation test was performed between plaque area and pro‐inflammatory markers, and a χ^2^ test was performed for incidence of papilloma virus infection. Significant differences were determined at *P <* 0.05. All data were analysed using GraphPad Prism 8.0 software (La Jolla, CA, USA).

## 3 RESULTS

### 3.1 Exercise capacity and body composition

Table 1 depicts the exercise capacity for the *ApoE* ^−/−^ Ex and *Tg26* ^+/−^ *ApoE* ^−/−^ Ex groups. Both groups showed significant increases in time to exhaustion and maximal velocity after physical training (*P* < 0.01). However, *ApoE* ^−/−^ Ex mice performed better than *Tg26* ^+/−^ *ApoE* ^−/−^ Ex mice at 4 weeks, as indicated by significant strain × time effects on both time to exhaustion (*P* = 0.02) and maximal velocity (*P* = 0.01).

**TABLE 1:** Exercise capacity after the experimental protocol (eighth week).

Parameter	*ApoE* ^−/−^ Ex	*Tg26* ^+/−^ *ApoE* ^−/−^ Ex	*P*‐value^a^		
	(*n* = 9)	(*n* = 5)	Strain	Time	Interaction
Time to exhaustion (min)					
Baseline	17.0 ± 4.8	15.4 ± 0.9			
4 weeks	25.9 ± 3.0	20.8 ± 0.8	0.12	<0.01	0.02
8 weeks	26.2 ± 2.7	25.6 ± 3.0			
Maximal velocity (m/min)					
Baseline	23.7 ± 4.4	21.8 ± 0.4			
4 weeks	30.9 ± 3.0	25.8 ± 0.8	0.10	<0.01	0.01
8 weeks	32.2 ± 2.7	31.6 ± 3.0			

*Note*: Values are presented as the mean ± SD.

^a^ Data were analysed by two‐way repeated‐measures ANOVA followed by Tukey's *post hoc* test for multiple comparisons.

**FIGURE 2:**
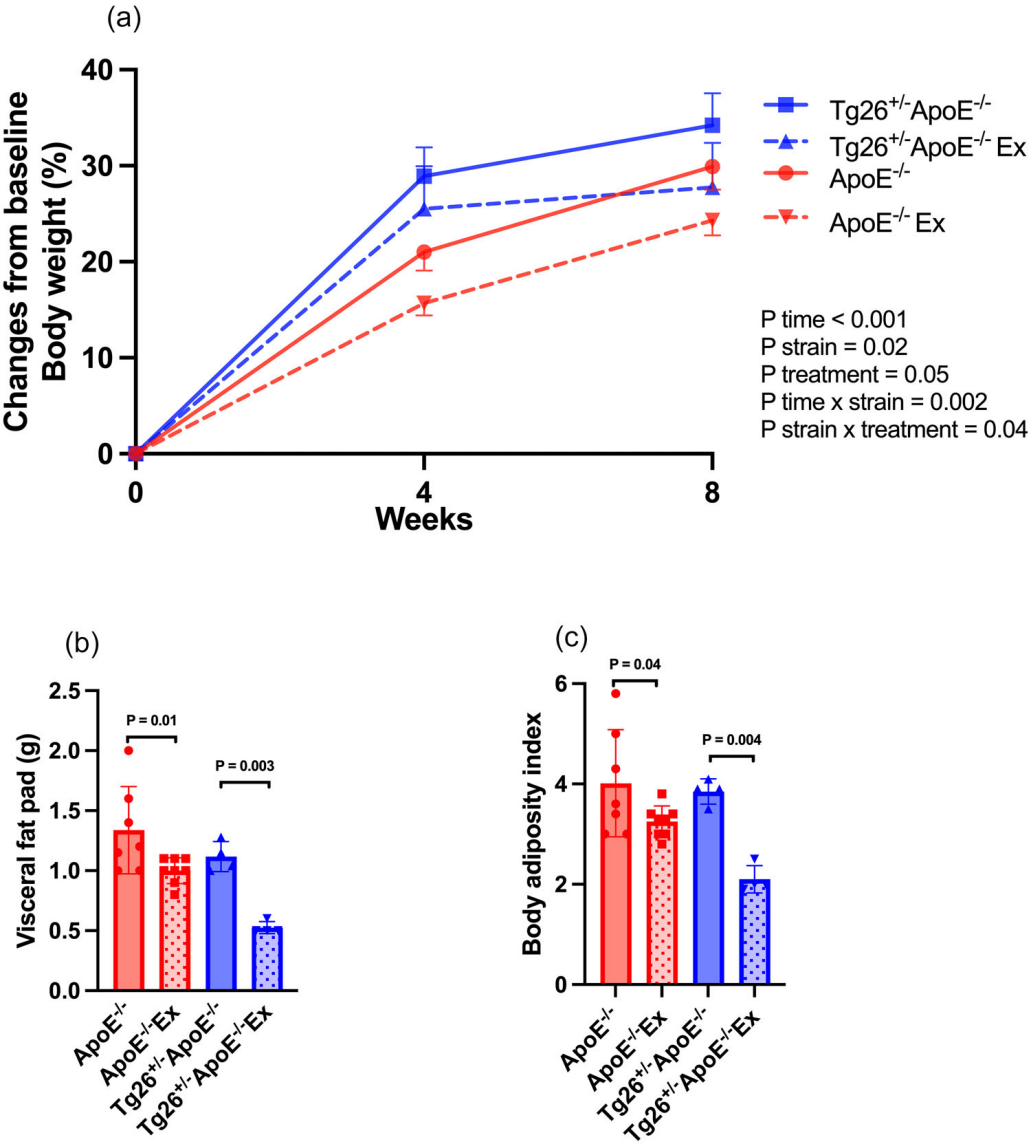
Effect of exercise training on body mass (a), visceral fat pad (b) and body adiposity index (c) in control and exercised *ApoE* ^−/−^ and *Tg26* ^+/−^ *ApoE* ^−/−^ mice. (a) All available animals from each group were included: *Tg26*⁺^/−^ *ApoE* ^−/−^ (*n* = 4), *ApoE* ^−/−^ (*n* = 9), *Tg26*⁺^/−^ *ApoE* ^−/−^ Ex (*n* = 5) and *ApoE* ^−/−^ Ex (*n* = 9). (b,c) Sample loss occurred during tissue harvesting and processing, resulting in the absence of two samples from the control *ApoE* ^−/−^ group and one sample each from the *ApoE* ^−/−^ Ex and *Tg26*⁺^/−^ *ApoE* ^−/−^ Ex groups. Data were analysed using three‐way (a) or two‐way (b,c) repeated‐measures ANOVA, followed by Tukey's *post hoc* test for multiple comparisons.

Figure 2 illustrates changes in body composition across all groups. At baseline, body mass was higher in *ApoE* ^−/−^ than *Tg26* ^+/−^ *ApoE* ^−/−^ mice (*ApoE* ^−/−^, 25.7 ± 1.4 g vs. *ApoE* ^−/−^ Ex, 27.4 ± 2.8 g vs. *Tg26* ^+/−^ *ApoE* ^−/−^, 21.7 ± 1.8 g vs. *Tg26* ^+/−^ *ApoE* ^−/−^ Ex, 19.8 ± 2.2 g, *P* = 0.0002; data not shown). Over time, body mass increased significantly in all groups (time *P* < 0.001; Figure 2a). However, *Tg26* ^+/−^ *ApoE* ^−/−^ mice exhibited a greater increase in body mass than *ApoE* ^−/−^ mice (time × strain *P* = 0.002; Figure 2a). Exercise effectively reduced the gain in body mass in both *ApoE* ^−/−^ Ex and *Tg26* ^+/−^ *ApoE* ^−/−^ Ex groups compared with their respective control groups (treatment *P* = 0.05; Figure 2a).

No differences were detected between non‐exercised groups for visceral fat pad (retroperitoneal, epididymal and inguinal) (*ApoE* ^−/−^, 1.3 ± 0.4 g vs. *Tg26* ^+/−^ *ApoE* ^−/−^, 1.1 ± 0.1 g; *P* = 0.6; Figure 2b) and body adiposity index (*ApoE* ^−/−^, 4.0 ± 1.1 vs. *Tg26* ^+/−^ *ApoE* ^−/−^, 3.9 ± 0.3; *P* = 0.9; Figure 2c). Exercise training significantly reduced visceral fat pad and body adiposity index in *Tg26* ^+/−^ *ApoE* ^−/−^ Ex (0.5 ± 0.1 g, *P* = 0.02 and 2.1 ± 0.3, *P* = 0.01, respectively; Figure 2b,c), but not in *ApoE* ^−/−^ Ex (1.0 ± 0.1 g, *P* = 0.06 and 3.2 ± 0.3 and *P* = 0.2, respectively; Figure 2b,c).

### 3.2 Aorta *en face* analysis

**FIGURE 3:**
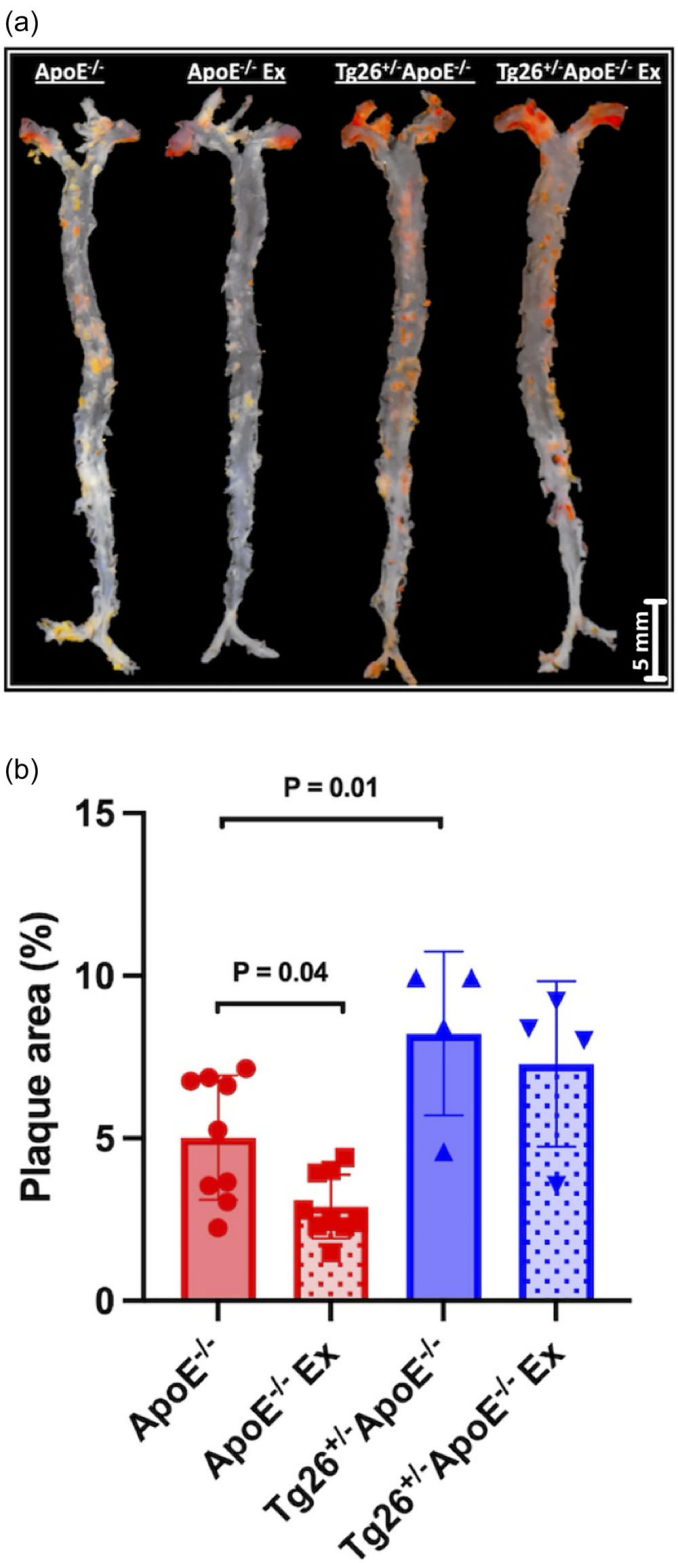
Representative *en face* aortas from each experimental group stained with Oil Red O to quantify plaque percentage from the aortic arch to the ileal bifurcation (a) and corresponding plaque area data (b) in control (*n* = 9) or exercised *ApoE* ^−/−^ (*n* = 9) and control (*n* = 4) or exercised *Tg26* ^+/−^ *ApoE* ^−/−^ mice (*n* = 4). Data are presented as the mean ± SD and analysed by two‐way repeated‐measures ANOVA followed by Tukey's *post hoc* test for multiple comparisons.

Figure 3 illustrates representative images and data for plaque area analysis. Atherosclerotic plaques were significantly larger in *Tg26* ^+/−^ *ApoE* ^−/−^ mice compared with *ApoE* ^−/−^ mice (8.22% ± 2.52% vs. 4.99% ± 1.61%, *P* = 0.01; Figure 3b). Exercise training significantly reduced plaque formation in *ApoE* ^−/−^ mice, as evidenced by a smaller plaque area in *ApoE* ^−/−^ Ex compared with *ApoE* ^−/−^ (2.95% ± 0.85% vs. 4.99% ± 1.61%, *P* = 0.04; Figure 3b). However, no significant difference in plaque area was observed between *Tg26* ^+/−^ *ApoE* ^−/−^ and *Tg26* ^+/−^ *ApoE* ^−/−^ Ex mice (8.22% ± 2.52% vs. 7.28% ± 2.54%, respectively, *P* = 0.85; Figure 3b).

### 3.3 Lipid profile

Table 2 illustrates the lipid profile, expressed by serum cholesterol and triglyceride concentrations across groups following exercise training. Significant strain effects were observed for both cholesterol (*P* = 0.02) and triglyceride levels (*P* = 0.02), with *Tg26* ^+/−^ *ApoE* ^−/−^ mice presenting overall lower cholesterol concentrations compared with *ApoE* ^−/−^ groups. A significant strain × treatment interaction was found for triglycerides (*P* = 0.04). Additionally, multiple comparisons revealed that *ApoE* ^−/−^ exercised mice showed significantly reduced triglyceride levels compared with *Tg26* ^+/−^ *ApoE* ^−/−^ exercised mice (*P* < 0.01).

**TABLE 2:** Lipid profile across groups after exercise training.

Parameter	*ApoE* ^−/−^	*ApoE* ^−/−^ Ex	*Tg26* ^+/−^ *ApoE* ^−/−^	*Tg26* ^+/−^ *ApoE* ^−/−^ Ex	*P*‐value^a^		
	(*n* = 5)	(*n* = 5)	(*n* = 3)	(*n* = 4)	Strain	Treatment	Interaction
Cholesterol (mg/dL)	1121.2 ± 188.7	1277.6 ± 228.5	900.6 ± 236.7	942.5 ± 262.6	**0.02**	0.39	0.61
Triglycerides (mg/dL)	116.4 ± 51.6	75.8 ± 14.8	122.0 ± 25.9	165.2 ± 43.6	**0.02**	0.94	**0.04**

*Note*: Values are presented as the mean ± SD. *P* values in bold indicate significant differences.

^a^ Data were analysed by two‐way repeated‐measures ANOVA followed by Tukey's *post hoc* test for multiple comparisons.

### 3.4 Kynurenine pathway, fresh blood immunophenotyping and cytokines

**FIGURE 4:**
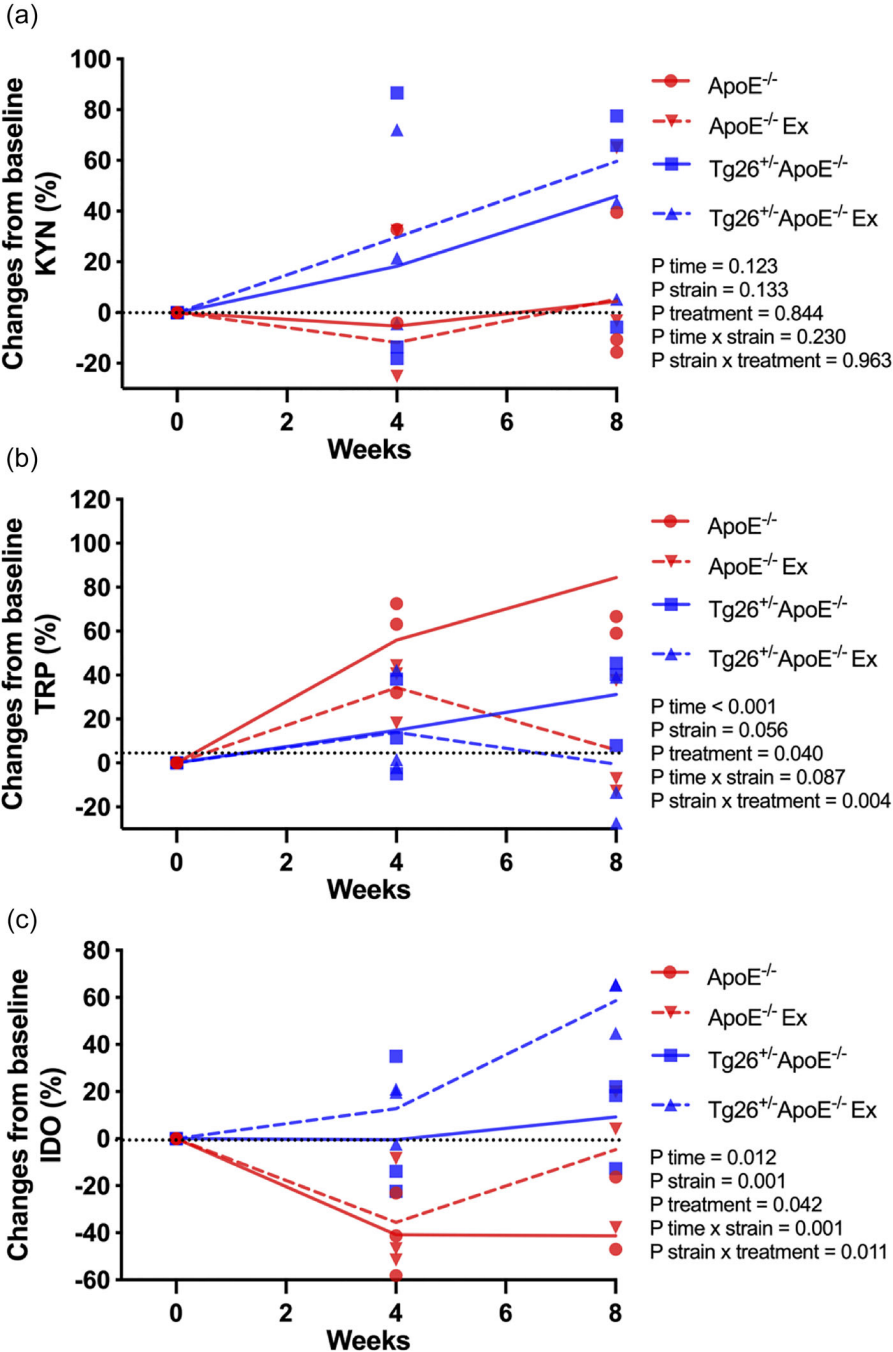
Changes from baseline in kynurenine (KYN; a), tryptophan (TRP; b) and indoleamine 2,3‐dioxygenase (IDO; c) concentrations in control (*n* = 4) or exercised *ApoE* ^−/−^ (*n* = 4) and control (*n* = 4) or exercised *Tg26* ^+/−^ *ApoE* ^−/−^ mice (*n* = 4) after exercise training. Each data point represents a repeated measurement from the same animal at different time points. Data were analysed using three‐way repeated‐measures ANOVA followed by Tukey's *post hoc* test for multiple comparisons.

Figure 4 illustrates changes in KYN pathway metabolites across groups over time. No differences were found in KYN levels between groups (*P* = 0.13 for strain effect and *P* = 0.84 for treatment effect; Figure 4a) or within groups over time (*P* = 0.12; Figure 4a). Regarding TRP kinetics, *ApoE* ^−/−^ mice exhibited increased TRP concentration over time (eighth week vs. baseline, *P* = 0.001; Figure 4b). Exercise training reduced TRP levels in *ApoE* ^−/−^ mice, because *ApoE* ^−/−^ Ex values were lower than those of *ApoE* ^−/−^ at the eighth week (*P* < 0.05; Figure 4b). No differences in TRP kinetics were detected between strains or within *Tg26* ^+/−^ *ApoE* ^−/−^ mice.

The IDO activity, expressed as the KYN/TRP ratio, was higher in *Tg26* ^+/−^ *ApoE* ^−/−^ than in *ApoE* ^−/−^ at the eighth week (*P* < 0.05; Figure 4c). Exercise training further increased IDO activity in *Tg26* ^+/−^ *ApoE* ^−/−^ mice, with *Tg26* ^+/−^ *ApoE* ^−/−^ Ex values being higher over time (eighth week vs. baseline and fourth week, *P* = 0.001; Figure 4c) and higher than those of *Tg26* ^+/−^ *ApoE* ^−/−^ mice at the eighth week (58.57% ± 6.88% vs. −4.62% ± 17.20%, *P* = 0.01; Figure 4c). No differences in IDO activity were detected within *ApoE* ^−/−^ mice.

Table 3 and Figure S1 present the inflammatory profile at the end of the experimental protocol (eighth week), and Table 4 presents their correlation (*R* values) with plaque area. Immunophenotyping showed that *Tg26* ^+/−^ *ApoE* ^−/−^ mice had slightly higher neutrophil (*P* = 0.05) and monocyte (*P* = 0.06) expression than *ApoE* ^−/−^ mice. No differences were detected in neutrophil (*P* = 0.97) or monocyte (*P* = 0.80) expression after exercise training. However, monocytes were positively related to plaque area in the *Tg26* ^+/−^ *ApoE* ^−/−^ group (*R* = 0.99, *P* = 0.01), but not in *Tg26* ^+/−^ *ApoE* ^−/−^ Ex mice (*R* = 0.56, *P* = 0.62). No relationships were found for neutrophils or monocytes in *ApoE* ^−/−^ groups.

**TABLE 3:** Immune–inflammatory profile at the end of experimental protocol (eighth week).

Parameter	*ApoE* ^−/−^	*ApoE* ^−/−^ Ex	*Tg26* ^+/−^ *ApoE* ^−/−^	*Tg26* ^+/−^ *ApoE* ^−/−^ Ex	Two‐way ANOVA (main effects)		
	(*n* = 4)	(*n* = 4)	(*n* = 4)	(*n* = 4)	Strain	Treatment	Interaction
Indoleamine 2,3‐dioxygenase (%)	−40.50 (18.34)	−4.59 (34.38)	16.67 (7.04)	50.00 (35.12)	*F* = 24.73 ***P*** **< 0.01**	*F* = 0.02 *P *= 0.87	*F* = 7.00 ***P *= 0.02**
Neutrophils (%)	21.75 (8.08)	26.75 (7.66)	36.77 (25.24)	41.30 (13.18)	*F* = 4.71 ***P** * **= 0.05**	*F* = 0.01 *P *= 0.97	*F* = 0.48 *P *= 0.49
Monocytes (%)	33.00 (9.70)	38.05 (5.94)	45.37 (23.22)	53.83 (14.80)	*F* = 4.44 *P *= 0.06	*F* = 0.06 *P *= 0.80	*F* = 1.02 *P *= 0.33
Interleukin‐6 (pg/mL)	39.27 (2.64)	43.24 (11.00)	41.76 (8.06)	52.38 (7.66)	*F* = 1.77 *P *= 0.21	*F* = 0.45 *P *= 0.51	*F* = 2.74 *P *= 0.12
Tumour necrosis factor‐α (pg/mL)	18.28 (4.80)	19.29 (9.72)	15.40 (13.60)	9.83 (8.76)	*F* = 0.85 *P *= 0.37	*F* = 1.15 *P *= 0.30	*F* = 0.01 *P *= 0.92
Interferon‐γ (pg/mL)	23.47 (2.88)	20.46 (1.00)	19.21 (3.46)	19.92 (3.18)	*F* = 1.67 *P *= 0.22	*F* = 0.62 *P *= 0.44	*F* = 0.01 *P *= 0.90

*Note*: Values are presented as the mean ± SD. *P* values in bold indicate significant differences.

**TABLE 4:** Relationship between plaque area and mean changes in immune–inflammatory profile at the end of the experimental protocol (eighth week).

Parameter	*R*(*P*‐value)			
	*ApoE* ^−/−^ (*n* = 4)	*ApoE* ^−/−^ Ex (*n* = 4)	*Tg26* ^+/−^ *ApoE* ^−/−^ (*n* = 4)	*Tg26* ^+/−^ *ApoE* ^−/−^ Ex (*n* = 4)
Indoleamine 2,3‐dioxygenase (%)	0.38 (0.61)	−0.38 (0.52)	0.99 (0.03)*	0.99 (0.02)*
Neutrophils (%)	0.15 (0.85)	−0.02 (0.97)	0.98 (0.10)	0.66 (0.53)
Monocytes (%)	0.23 (0.76)	−0.02 (0.97)	0.99 (0.01)*	0.56 (0.62)
Interleukin‐6 (pg/mL)	−0.41 (0.58)	0.64 (0.24)	0.99 (0.08)	0.15 (0.90)
Tumour necrosis factor‐α (pg/mL)	−0.64 (0.35)	0.58 (0.30)	−0.91 (0.26)	−0.99 (0.05)*
Interferon‐γ (pg/mL)	0.51 (0.48)	0.40 (0.49)	−0.23 (0.84)	−0.98 (0.09)

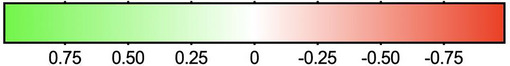
.

*P* values are presented in parentheses.

Additionally, Table 4 shows that IDO activity was positively related to plaque area in both *Tg26* ^+/−^ *ApoE* ^−/−^ mice (*R* = 0.99, *P* = 0.03) and *Tg26* ^+/−^ *ApoE* ^−/−^ Ex mice (*R* = 0.99, *P* = 0.02), but not in *Tg26* ^+/−^ *ApoE* ^−/−^ Ex mice (*P* = 0.61 and *P* = 0.52, respectively).

No significant differences were found for pro‐inflammatory cytokine levels between groups or correlation with plaque area in any group, except for *Tg26* ^+/−^ *ApoE* ^−/−^ Ex mice, in which TNF‐α was negatively related to plaque area (*R* = −0.99, *P* = 0.05; Table 4).

## 4 DISCUSSION

The aim of this study was to investigate the effect of aerobic exercise training on the development of HIV‐associated atherogenesis and TRP catabolism via the KYN pathway in an HIV‐associated atherosclerosis mouse model. Key findings include: (1) moderate‐intensity aerobic training reduced plaque area in *ApoE* ^−/−^ mice, but not in HIV transgenic mice; and (2) exercise training appeared to increase inflammation in HIV transgenic mice, as indicated by elevated IDO activity.

We also observed accelerated plaque development in control HIV transgenic mice, accompanied by heightened IDO activity and increased levels of circulating monocytes and neutrophils. Notably, the accelerated plaque development in this mouse model is correlated with IDO activity and is not influenced by serum triglycerides or cholesterol, as previously demonstrated by our group (Kearns, Velasquez, Liu, et al., 2019). Other inflammatory mechanisms seem to be associated with plaque development in HIV transgenic mice (Kearns et al., 2017). Importantly, NLRP3 inflammasone‐mediated caspase‐1, traditionally linked to CD4 T‐cell depletion by pyroptosis, contributes to both cell death (Doitsh et al., 2014) and atherosclerosis in HIV infection, with its deficiency attenuating atherosclerosis development in HIV transgenic mice (Alam et al., 2023; Kearns, Liu, Dai, et al., 2019). The heightened inflammatory environment related to HIV might have influenced the potential benefits of exercise training on atherosclerosis.

Aerobic exercise training, particularly at moderate intensity, mitigates the progression of atherosclerotic plaques in both humans and *ApoE* ^−/−^ mice by improving lipid profiles, enhancing endothelial function and reducing systemic inflammation (Meyer‐Lindemann et al., 2023). Importantly, our lipid profile data revealed strain‐ and training‐related differences in cholesterol and triglyceride levels. Although *ApoE* ^−/−^ mice showed overall higher cholesterol levels than *Tg26* ^+/−^ *ApoE* ^−/−^ mice, they had greater responses than *Tg26* ^+/−^ *ApoE* ^−/−^ mice to exercise training, as indicated by significant strain × treatment effects on triglyceride levels. These findings suggest that although both genotypes share a similar dyslipidaemic baseline attributable to ApoE deficiency, the lipid‐lowering effect of exercise might be blunted in the HIV‐transgenic model. This impaired response could be attributed to persistent inflammation or reduced metabolic flexibility in the presence of HIV‐related immune activation, reinforcing the notion that the attenuated effect of exercise on plaque burden in HIV mice is not mediated solely by traditional cardiometabolic factors.

In our study, improvements in body mass, body composition and exercise capacity observed in both genotypes are consistent with previous findings linking these benefits to various inflammatory and metabolic mechanisms (Huang et al., 2022; Li et al., 2022, 2020). Although we selected aerobic training to isolate its specific effects on atherosclerosis and TRP catabolism, it is important to acknowledge that combined training (aerobic plus strength) might offer additional benefits for certain outcomes, such as cardiorespiratory fitness and lean body mass. Nevertheless, these improvements might not necessarily translate into superior effects on cardiometabolic biomarkers when compared with aerobic training alone (Terada et al., 2024). To date, no studies have investigated the effects of any type of exercise training on atherosclerosis and the immunoinflammatory profile in HIV settings. Our findings help to fill this gap by showing that moderate aerobic training did not reduce the atherosclerotic plaque area in an HIV‐associated atherosclerosis mouse model, despite improvements in body composition and exercise capacity. This absence of effect was accompanied by increased IDO activity in the exercised HIV transgenic mice, and plaque area was positively correlated with IDO activity in these mice, suggesting that the inflammatory environment might have attenuated the potential protective effects of exercise.

IDO plays a dual role in inflammation. It modulates the immune response by depleting TRP and promoting the formation of metabolites that can suppress T‐cell proliferation and induce regulatory T cells, thereby controlling excessive inflammation and promoting immune tolerance (Grishanova & Perepechaeva, 2024). However, when chronically activated, IDO can contribute to chronic inflammation and exacerbate diseases such as atherosclerosis through pro‐inflammatory metabolites (Grishanova & Perepechaeva, 2024). Our findings suggest that exercise training affected HIV transgenic mice differently from *ApoE* ^−/−^ mice owing to further increases in IDO activity, which probably amplified inflammation (Kearns, Velasquez, Liu, et al., 2019). These findings highlight the complexity of the role of exercise in HIV‐associated atherosclerosis and suggest that a tailored approach might be necessary when recommending exercise for people living with HIV.

Exercise training induces molecular adaptations in immune function, primarily through secretion of myokines by skeletal muscles (Pedersen & Febbraio, 2012). It can trigger metabolic changes in macrophages, promoting an anti‐inflammatory phenotype by increasing PPAR‐γ mRNA expression. This enhances T‐cell metabolism by improving T‐cell receptor activation signals and boosting Zap70 expression (Rosa‐Neto et al., 2022). However, the immune response to exercise is influenced by its type, intensity and duration, and overtraining can worsen the immune‐inflammatory profile (Lakier Smith, 2003; Rosa‐Neto et al., 2022). In the context of HIV, viral transcripts might make these mice more prone to overexertion (Kearns et al., 2017). Our data on IDO activity support this premise. Additionally, although there were no differences in body mass and exercise capacity between groups, exercised HIV transgenic mice showed greater irritability during exercise sessions and a higher incidence of mouse papillomavirus infection than control HIV transgenic and *ApoE* ^−/−^ mice (60% vs. 0% in *Tg26* ^+/−^ *ApoE* ^−/−^ and *ApoE* ^−/−^, *P* = 0.01; data not shown). Evidently, this hypothesis warrants further investigation.

The exercise protocol used in this study was well tolerated by *ApoE* ^−/−^ mice, showing benefits consistent with existing literature (Jakic et al., 2019; Kadoglou et al., 2022; Kim et al., 2020). Notably, exercise training decreased TRP levels in *ApoE* ^−/−^ mice. The literature suggests that acute exercise might enhance the conversion of TRP into KYN via IDO, probably owing to a transient increase in inflammation (Joisten et al., 2020). Over time, regular exercise appears to shift the KYN pathway from producing its neurotoxic and pro‐inflammatory metabolite, quinolinic acid, towards producing kynurenic acid (KA) (Cervenka et al., 2017), which is the protective branch. Agudelo et al. (2019) found that this redirection is likely to be mediated by an increase in expression of the enzyme kynurenine aminotransferase, driven by activation of peroxisome proliferator‐activated receptor gamma coactivator‐1 during exercise, which promotes the conversion of KYN to KA. In turn, KA might influence immune function by promoting the differentiation of T helper 17 cells into regulatory T cells through its activity as an Aryl Hydrocarbon Receptor (AhR) agonist. Additionally, KA might facilitate KYN clearance in the periphery, contributing to an anti‐inflammatory state via Gpr‐35 activation (Agudelo et al., 2018; Joisten et al., 2020). Data on further metabolites of the KYN pathway, such as KA and quinolinic acid, would help to provide a better understanding of the effects of exercise training on the KYN pathway and its relationship to atherosclerosis, which deserves future studies (Baumgartner et al., 2019).

Aside from the negative correlation between plaque size and TNF‐α, expression levels of neutrophils, monocytes and pro‐inflammatory cytokines remained unchanged after exercise training. The large but non‐significant difference in IL‐6 levels between exercised and control HIV transgenic mice suggest that the study might have been underpowered to detect this end‐point owing to the relatively small sample size. Although evidence suggests a potential direct contribution of cytokines such as TNF‐α and IFN‐γ to development of atherosclerosis (Bouchareychas et al., 2020; Sudar‐Milovanovic et al., 2022), previous studies have also shown exercise‐induced reduction in plaque size in *ApoE* ^−/−^ animals without changes in inflammatory markers (Kadoglou et al., 2022; Stanton et al., 2022). The *ApoE* ^−/−^ mouse is a classic model for atherosclerosis owing to its capacity for development of hyperlipidaemia (Bouchareychas et al., 2020), and the reduction in plaque size might be mediated through exercise‐induced changes in the lipid profile, as supported by several studies (Muscella et al., 2020).

This study has strengths and limitations. A major limitation is that the lipid profile, expression levels of monocytes neutrophils, and cytokines could be assessed in only a subset of mice at the end of the experiment owing to limited availability of blood at baseline and the midpoint, limiting inferences of cause and effect over time. Also, marked physiological differences are observed between different species; thus, direct extrapolation of these findings from mice to humans should be approached with caution. Despite this limitation, this study represents the first step towards understanding the effect of exercise training on dynamics of TRP metabolism in HIV‐associated ASCVD. The innovative nature of this research advances our understanding of non‐pharmacological therapeutic strategies for managing HIV‐associated ASCVD, underscoring its significance.

## 5 CONCLUSION

In conclusion, although aerobic moderate‐intensity training reduced plaque area in *ApoE* ^−/−^ mice, it did not have the same effect in HIV‐associated atherosclerosis mice. Instead, exercise training appeared to increase inflammation in *Tg26* ^+/−^ *ApoE* ^−/−^ mice, as evidenced by elevated IDO activity. The HIV‐associated atherosclerosis model might heighten susceptibility to overexertion and overtraining. Further investigation is needed to elucidate the dose–response relationship between aerobic training and markers of atherosclerosis and inflammation in HIV contexts.

## AUTHOR CONTRIBUTIONS

Marcus Rangel: Conceptualization, methodology, data curation, formal analysis, and original draft preparation. Mohammad Afaque Alam, Mohammad Islamuddin, and Zheng Chen: Methodology, writing – reviewing and editing. Xuebin Qin: Conceptualization, project administration, supervision, resources, funding acquisition, and writing – reviewing and editing. Juliana Borges: Conceptualization, original draft preparation, supervision, and funding acquisition. All authors read and approved the final version of the manuscript and agree to be accountable for all aspects of the work in ensuring that questions related to the accuracy or integrity of any part of the work are appropriately investigated and resolved. All persons designated as authors qualify for authorship, and all those who qualify for authorship are listed.

## CONFLICT OF INTEREST

None declared.

## Supporting information



Supporting Information

## Data Availability

The data that support the findings of this study will be made available upon reasonable request to the corresponding author.
