# Supplementary figures and images for: Exercise‐induced effects on atherogenesis and tryptophan catabolism via the kynurenine pathway in an HIV‐associated atherosclerosis mouse model
Source: Exp Physiol. 2025 Aug 25;111(3):866–76. doi: 10.1113/EP092744 (PMC12949122; doi:10.1113/EP092744)

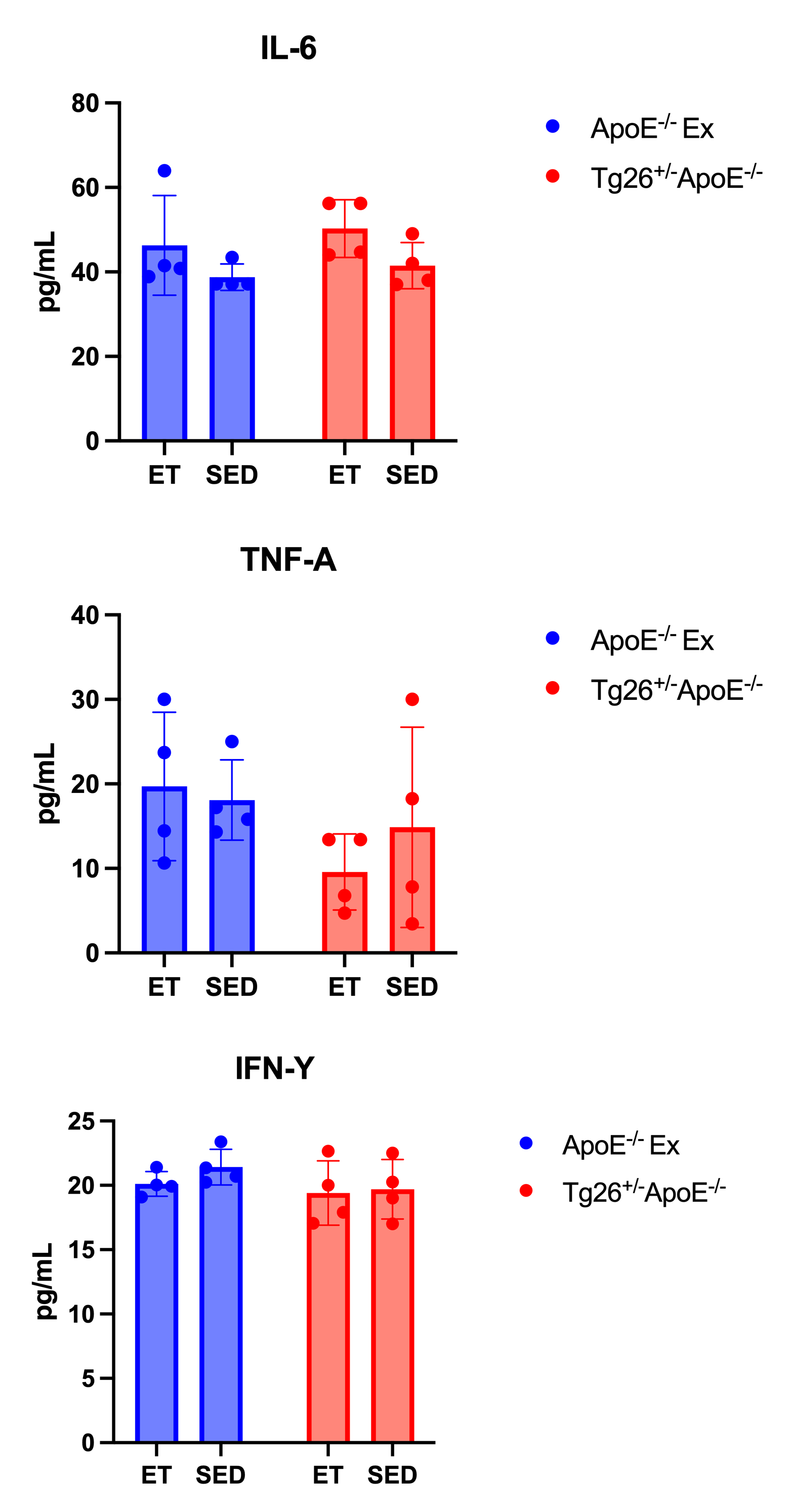

Supplement: Supplementary file 1 — Supporting Information [file EPH-111-866-s001.tif]
